# *DRD4* Exon 3 Gene Polymorphisms in Patients Diagnosed with Polysubstance Use Disorder and Co-Occurrence of a Depressive Episode

**DOI:** 10.3390/genes12111834

**Published:** 2021-11-20

**Authors:** Krzysztof Chmielowiec, Jolanta Chmielowiec, Jolanta Masiak, Małgorzata Czekaj, Piotr Krawczyk, Ewelina Soroka, Małgorzata Śmiarowska, Wojciech Musiał, Tomasz Pawłowski, Anna Grzywacz

**Affiliations:** 1 Department of Hygiene and Epidemiology, Collegium Medicum, University of Zielona Góra, 28 Zyty St., 65-046 Zielona Góra, Poland; chmiele@vp.pl (K.C.); chmiele1@o2.pl (J.C.); 2 Second Department of Psychiatry and Psychiatric Rehabilitation, Medical University of Lublin, 1 Głuska St., 20-059 Lublin, Poland; jolanta.masiak@umlub.pl (J.M.); ewelina.soroka@umlub.pl (E.S.); 3 Department of Nervous System Diseases, Orthopedics, Traumatology and Oncology of the Locomotor System, Collegium Medicum, University of Zielona Góra, 28 Zyty St., 65-046 Zielona Góra, Poland; vielgo@wp.pl (M.C.); krawczykp@poczta.onet.pl (P.K.); wojamus@wp.pl (W.M.); 4 Department of Pharmacokinetics and Therapeutic Drug Monitoring, Pomeranian Medical University, Aleja Powstancόw Wlkp. 72 St., 70-111 Szczecin, Poland; malgorzata.smiarowska@wp.pl; 5 Division of Psychotherapy and Psychosomatic Medicine, Wroclaw Medical University, Wyb. L. Pasteura 10 St., 50-367 Wroclaw, Poland; tomasz.pawlowski@umed.wroc.pl; 6 Independent Laboratory of Health Promotion, Pomeranian Medical University in Szczecin, 11 Chlapowskiego St., 70-204 Szczecin, Poland

**Keywords:** addiction, dual diagnosis, *DRD4* gene

## Abstract

Background: There has been a noticeable and systematic growth of the use of psychoactive substances over the past few decades. Dual diagnosis is a clinical term referring to the occurrence of psychoactive substance use disorder comorbid with another psychiatric disorder in the same person. The most common type of dual diagnosis is the co-occurrence of alcohol use disorder and mood disorders in the form of a depressive episode. Co-occurrent substance use disorders are frequently influenced by genetic factors. In selecting our area of research, we focused on dopamine and the *DRD4* (Dopamine Receptor D4) gene polymorphism as well as associations with personality features. The aim of the study: The aim of the study was to compare *DRD4* exon 3 (DRD4 Ex3) gene polymorphisms in patients diagnosed with polysubstance use disorder and co-occurrence of a depressive episode to *DRD4* exon 3 gene polymorphisms in patients diagnosed with polysubstance use disorder and without co-occurrence of a depressive episode and a group of healthy volunteers. The study also aimed at establishing associations between personality features and *DRD4* exon 3 gene polymorphisms of male patients diagnosed with polysubstance use disorder with co-occurrence of a depressive episode which may present a specific endophenotype of this group of patients. Methods: The study group comprised 602 male volunteers: patients diagnosed with polysubstance use disorder comorbid with a depressive episode (PUD MDD) (*n* = 95; mean age = 28.29, standard deviation (SD) = 7.40), patients diagnosed with polysubstance use disorder (PUD) (*n* = 206; mean age = 28.13, SD = 5.97), and controls (*n* = 301; mean age = 22.13, SD = 4.57). The patients and control subjects were diagnosed by a psychiatrist using the Mini International Neuropsychiatric Interview (MINI), the NEO Five-Factor Personality Inventory (NEO-FFI), and the State-Trait Anxiety Inventory (STAI) questionnaires. An analysis of the *DRD4* exon 3 polymorphism was performed. Results: The patients diagnosed with PUD MDD compared to the control group of healthy volunteers showed significantly higher scores on both the STAI status and features scale and the NEO-FFI Neuroticism and Openness Scale, as well as lower scores on the Extraversion, Agreeableness, and Conscientiousness NEO-FFI scales. In the *DRD4* exon 3 gene polymorphism, the s allele was more frequent in the PUD MDD compared to the l allele, which was less frequent. The results of the 2 × 3 factor analysis of variance (ANOVA) in patients and controls and the variant *DRD4* exon 3 interaction were found on the Extraversion Scale and the Conscientiousness Scale of the NEO-FFI. Conclusions: The associations show that psychological factors combined with genetic data create a new area of research on addiction, including the problem of dual diagnosis. However, we want to be careful and draw no definite conclusions at this stage of our research.

## 1. Introduction

There has been a noticeable and systematic growth of the use of psychoactive substances over the past few decades [1]. Dual diagnosis is a clinical term referring to the occurrence of psychoactive substance use disorder comorbid with another psychiatric disorder in the same person [1]. The literature reports that approximately 50.9% of patients with psychiatric disorders are also diagnosed with psychoactive substance use disorder or alcohol use disorder [1]. Co-occurrent disorders are more frequently reported amongst males than females [2]. The most common type of dual diagnosis is the co-occurrence of alcohol use disorder and mood disorders in the form of a depressive episode [1]. Depression occurs 3–4 times more often in people with substance use disorders than in healthy controls [2,3]. If a depressive episode is severe, it largely anticipates faster development and maintenance of substance use disorder [2]. The occurrence of dual diagnosis in patients makes the treatment process more difficult both in terms of pharmacotherapy and psychotherapy [2] and may also contribute to a more frequent occurrence of suicidality. 

Therefore, our study focused on a group of patients suffering from polysubstance use disorder who also had comorbid diagnosis of a depressive episode, and the study aimed at comparing *DRD4* exon 3 gene polymorphisms in patients diagnosed with polysubstance use disorder and co-occurrence of a depressive episode to *DRD4* exon 3 gene polymorphisms in patients diagnosed with polysubstance use disorder and without co-occurrence of a depressive episode, and a group of healthy volunteers. The study also aimed at establishing associations between personality features and *DRD4* exon 3 gene polymorphisms of male patients diagnosed with polysubstance use disorder with co-occurrence of a depressive episode, which may present a specific endophenotype of this group of patients.

Co-occurrent substance use disorders are frequently influenced by genetic factors [4]. In selecting our area of research, we focused on dopamine and the *DRD4* gene (dopamine receptor D4 gene). 

Several clinical genetic linkage studies demonstrate possible correlations between DRD4 expression and substance use disorders, as well as depression [5]. Carriers of the *DRD4* 7R allele showed greater susceptibility to alcohol use disorder and opioid use disorder [5]. The inconsistency in studies on relations between *DRD4* polymorphisms and dependencies suggests focusing on addiction-related phenotypes rather than on a diagnosis of dependency itself. 

The *DRD4* gene is located in chromosome 11p, close to the telomere. It encodes the 7-transmembrane G protein-coupled receptor that responds to endogenous dopamine [6,7,8]. The exon 3 seven-repeat (7R) allele of *DRD4* codes for a decreased dopamine receptor affinity and is associated with frequent substance abuse [9].

The existing research on the role of the *DRD4* (VNTR) polymorphism for somebody’s personality is inconclusive [10]. The allelic variation in the *DRD4* dopamine gene has been associated with novelty seeking [10] and impulsivity [11]. These results thus confirmed previous findings in which the long repeats of the *DRD4* exon 3 polymorphism were related to the novelty-seeking personality trait. In the existing research, attempts are made to recognize associations between the *DRD4* (VNTR) polymorphism and extraversion [12,13,14,15,16]. In a comprehensive metanalysis, substance use disorder was associated with high disinhibition, low conscientiousness and low agreeableness, but it was not significantly associated with neuroticism and extraversion [17]. The lack of knowledge in the field of personality features of patients diagnosed with polysubstance use disorder and cooccurrence of a depressive episode as well as associations of these factors with *DRD4* exon 3 gene polymorphisms inspired us to undertake this research.

## 2. Materials and Methods

### 2.1. Materials

The study group of 602 male volunteers comprised patients diagnosed with polysubstance use disorder comorbid with a depressive episode (PUD MDD; *n* = 95; mean age = 28.29, SD = 7.40), patients diagnosed with polysubstance use disorder (PUD; *n* = 206; mean age = 28.13, SD = 5.97) and healthy controls (*n* = 301; mean age = 22.13, SD = 4.57). The distribution of particular types of substance use disorder in the study group is presented as percentages in Table 1. After the approval of the Bioethics Committee of the Pomeranian Medical University in Szczecin (KB-0012/106/16) and when the written informed consent of the participants had been gained, the study was carried out in the Independent Laboratory of Health Promotion. After at least three months of abstinence in addiction treatment facilities, the patients with polysubstance use disorder (PUD) and patients with polysubstance use disorder comorbid with a depressive episode (PUD MDD) were recruited for the study. The patients with polysubstance use disorder (PUD) and polysubstance use disorder comorbid with a depressive episode (PUD MDD) as well as the control subjects were interviewed by a psychiatrist using the Mini International Neuropsychiatric Interview (MINI), the NEO Five-Factor Personality Inventory (NEO-FFI), and the State-Trait Anxiety Inventory (STAI).

Interactions between personality traits and *DRD4* exon 3 gene polymorphisms were examined only for the group of patients diagnosed with PUD MDD and non-dependent controls.

### 2.2. Measures

The MINI is a structured diagnostic interview, developed to assess the diagnoses of psychiatric patients according to DSM-IV and ICD-10 criteria. In our investigation, the study group and control subjects were examined by a psychiatrist using the MINI.

The STAI measures anxiety as a trait of anxiety (A-Trait) that can be described as an enduring predisposition to having worries, stress, and discomfort and anxiety states (A-states), such as uneasiness, fear, and temporary stimulation of the autonomic nervous system in response to certain circumstances. 

The Personality Inventory (NEO Five-Factor Inventory, NEO-FFI) incorporates 6 components for each of the five traits—neuroticism (anxiety, hostility, depression, self-consciousness, impulsiveness, vulnerability to stress), extraversion (warmth, gregariousness, assertiveness, activity, excitement seeking, positive emotion), openness to experience (fantasy, aesthetics, feelings, actions, ideas, values), agreeableness (trust, straightforwardness, altruism, compliance, modesty, tendermindedness), and conscientiousness (competence, order, dutifulness, achievement striving, self-discipline, deliberation) [18]. 

The results delivered by the inventories, i.e., NEO-FFI and STAI, were returned as sten scores. For the conversion of raw results into the sten scale scores, which was performed according to the Polish norms regarding adults, it was assumed that 1–2 accounted for very low scores, 3–4 accounted for low scores, 5–6 accounted for average scores, 7–8 accounted for high scores, and 9–10 accounted for very high scores.

### 2.3. Genotyping

Tubes containing EDTA (anticoagulant) were used for collecting blood for genetic assays. Genomic DNA from blood leukocytes was obtained using the High Pure Polymerase Chain Reaction (PCR) Template Preparation extraction kit (Roche Diagnostics, Mannheim, Germany). The process of extraction was performed according to the manufacturer’s instructions. The extracted samples of DNA had been stored at 4°C before further analysis was carried out.

The genomic DNA was sourced from venous blood drawn according to standard procedures. In order to genotype the samples, the PCR method was used. The *DRD4* genotypes were grouped based on the presence of the short (2–5 repeat) and long (6–11 repeat) variants. Genotyping was conducted using the PCR-VNTR method and included the following primers: F: 5 0-GCG ACT ACG TGG TCT ACT CG 3 0, R: 5 0-AGG ACC CTC ATG GCC TTG 3 0; in the final volume of 25 μL PCR mix per reaction, with l00 ng of genomic DNA, 10 pmol of primers, 50 mM KCl, 10 mM TrisHCl, 1.5 mM MgCl2, 200 μM dATP, dCTP, dTTP, dGTP, and 0.8 U of the Tag polymerase. The reaction occurred under the following conditions: 3 min of initial denaturation at 95 °C, cycles of denaturation at 95 °C for 30 s, hybridization of primers at 63 °C for 1 min, and elongation at 72 °C for 30 s, repeated in 35 cycles, with final elongation at 72 °C for 5 min. The amplified products were visualized using ethidium bromide-stained gel electrophoresis (3% agarose) and UV photography. The products ranged from 379 bp (2 repeats) to 811 (11 repeats) and were divided into 2 groups: short alleles (S, 2–5 repeats) and long alleles (L, 6–11 repeats).

### 2.4. Statistical Analysis

Concordance between the genotype frequency distribution and Hardy–Weinberg equilibrium (HWE) was verified using the HWE software (https://wpcalc.com/en/equilibrium-hardy-weinberg/ (20 March 2021)). The relationships between *DRD4* exon 3 (*DRD4* Ex3) variants, PUD, PUD MDD, control subjects, and the NEO Five Factor Inventory (NEO-FFI) were analyzed using a multivariate analysis of factor effects ANOVA [NEO-FFI/scale STAI/ × genetic feature × control and PUD and PUD MDD × (genetic feature × control and PUD/PUD MDD)]. The condition of homogeneity of variance was fulfilled (Levene test *p* > 0.05). The analyzed variables were not distributed normally. The NEO Five Factor Inventory (neuroticism, extraversion, openness, agreeability, and conscientiousness) was applied and compared using the Mann–Whitney U-test. *DRD4* exon 3 genotype frequencies between the healthy control subjects and PUD MDD and PUD subjects were established using the chi-square test. All computations were made using STATISTICA 13 (Tibco Software Inc., Palo Alto, CA, USA) for Windows (Microsoft Corporation, Redmond, WA, USA).

## 3. Results

For the patients diagnosed with PUD MDD, PUD, and control subjects, the frequency distributions accorded with the HWE (Table 2).

No difference for *DRD4* exon 3 (Ex3) genotype frequencies in the sample group was found between patients diagnosed with PUD MDD, patients diagnosed with PUD, and control subjects (Table 3).

The means and standard deviations for all the NEO Five Factor Inventory results and the STAI scale state and trait scale variant interactions for the PUD MDD and PUD and control subjects are presented in Table 4.

Compared to the control group, no statistically significant difference in the genotype frequency for the *DRD4* exon 3 (Ex3) gene in the patients diagnosed with PUD MDD and patients diagnosed with PUD was found. A statistically significant difference was found in the frequency of *DRD4* exon 3 (Ex3) alleles between all patients diagnosed with PUD MDD and the control group (s 0.82 vs. 0.75, l 0.18 vs. 0.25, χ^2^ = 3.99, *p* = 0.046). 

While comparing the controls and the PUD MDD subjects, for the latter, we found significantly higher scores on the STAI trait scale (M 7.62 vs. M 5.16, *p* < 0.001), the STAI state scale (M 6.65 vs. M 4.68, *p* < 0.001), the NEO Five Factor Inventory Scale of Neuroticism (M 7.34 vs. M 4.67, *p* < 0.001), and the NEO Five Factor Inventory Scale of Openness (M 5.46 vs. M 4.53, *p* < 0.001).

Compared to the controls, the case group subjects had significantly lower scores on the NEO Five Factor Inventory Scale of Extraversion (M 5.44 vs. M 6.37, *p* < 0.001), the NEO Five Factor Inventory Scale of Agreeability (M 4.05 vs. M 5.60, *p* < 0.001), and the NEO Five Factor Inventory Scale of Conscientiousness (M 5.01 vs. M 6.07, *p* < 0.001).

Regarding the controls and PUD subjects, for the latter, we found significantly higher scores on the STAI trait scale (M 6.87 vs. M 5.16, *p* < 0.001), the STAI state scale (M 5.54 vs. M 4.68, *p* < 0.001), and the NEO Five Factor Inventory Scale of Neuroticism (M 6.45 vs. M 4.67, *p* < 0.001).

Compared to the controls, the case group subjects had significantly lower scores on the NEO Five Factor Inventory Scale of Extraversion (M 5.90 vs. M 6.37, *p* = 0.0147) and the NEO Five Factor Inventory Scale of Agreeability (M 4.41 vs. M 5.60, *p* < 0.001).

Regarding the PUD MDD subjects and PUD subjects, for the latter, we found significantly higher scores on the STAI trait scale (M 7.62 vs. M 6.87, *p* = 0.0002), the STAI state scale (M 6.65 vs. M 5.54, *p* = 0.0059), and the NEO Five Factor Inventory Scale of Neuroticism (M 7.34 vs. M 5.85, *p* = 0.002).

Compared to the controls, the case group subjects had significantly lower scores on the NEO Five Factor Inventory Scale of Conscientiousness (M 5.01 vs. M 6.37, *p* = 0.0027).

The results of the 2 × 3 factorial ANOVA of the NEO Five-Factor Personality Inventory (NEO–FFI) and the State-Trait Anxiety Inventory (STAI) sten scales are summarized in Table 5 and Table 6.

No statistically significant differences were found between the groups (PUD/PUD MDD vs. controls) on the NEO FFI scale, or alleles s and l *DRD4* exon 3 (Ex3).

No statistically significant differences were found between the groups (PUD vs. controls) on the NEO FFI Extraversion Scale and the polymorphism of the *DRD4* exon 3 (Ex3) gene. 

**Figure 1:**
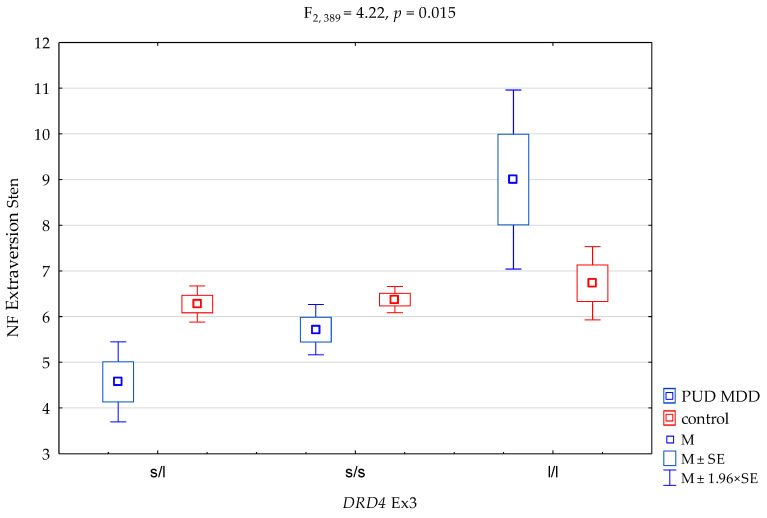
Interaction between patients with polysubstance use disorder comorbid with a depressive episode (PUD MDD)/control and *DRD4* exon 3 (*DRD4* Ex3) and the NEO FFI Extraversion Scale. M, mean; SE, standard error.

We received a significant result for *DRD4* exon 3 (Ex3) (F_2,389_ = 6.23, *p* = 0.002) on the NEO-FFI Extraversion Scale, which accounted for 3.1% of the variance. With regard to interactions, we found a significant result for the groups (PUD MDD vs. controls) on the NEO FFI Extraversion Scale, and *DRD4* exon 3 (Ex3) (Figure 1, F_2,389_ = 4.22, *p* = 0.015) accounted for 2.1% of the variance (Table 5). The results of the post hoc test are shown in Table 7.

**Figure 2:**
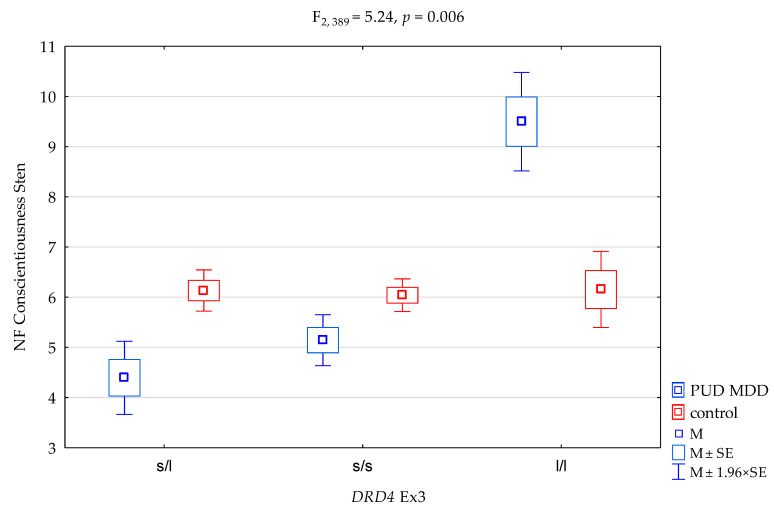
Interaction between patients with polysubstance use disorder comorbid with a depressive episode (PUD MDD)/control and *DRD4* exon 3 (*DRD4* Ex3) and the NEO FFI Conscientiousness/scale. M, mean; SE, standard error.

We found a significant result for *DRD4* exon 3 (Ex3) (F_2,389_ = 5.09, *p* = 0.007) on the NEO-FFI Conscientiousness Scale, which accounted for 2.6% of the variance. With regard to interactions, we received a significant result for the groups (PUD MDD vs. controls) on the NEO FFI Conscientiousness Scale, and *DRD4* exon 3 (Ex3) (Figure 2, F_2,389_ = 5.24, *p* = 0.006) accounted for 2.6% of the variance (Table 5). The results of the post hoc test are shown in Table 7.

## 4. Discussion

Compared to the controls, we found no statistically significant differences in the genotype frequency for the *DRD4* exon 3 (Ex3) gene in the patients diagnosed with PUD MDD and patients diagnosed with PUD.

Overall, a vast variety of clinical genetic linkage studies have explored possible correlations between *DRD4* expression and addiction. A study carried out by Shao et al. revealed that a significantly greater cue-induced craving for heroin was found in heroin-dependent Chinese subjects carrying the *DRD4* VNTR long-type allele, compared to nondependent controls [19]. A study by Ray et al. revealed a significant, direct path between the *DRD4* VNTR genotype and alcohol abuse [20]. A more recent study revealed that the homozygous repeat 7R/7R of *DRD4* was significantly associated with substance abuse disorder in Jordan’s population [21].

Furthermore, we ask ourselves whether depression is also genetically linked to substance use disorder, especially in the context of the *DRD4* gene and its VNTR polymorphic variant in exon 3. A considerable number of studies indicate that there is a link between depression and the dopaminergic system. On the one hand, a meta-analysis of 12 studies of VNTR of the *DRD4* gene polymorphism with depression performed by López León et al. (2005) revealed that the short allele 2 is associated with depression [22], but on the other hand, Gafarov et al. [23] found that male carriers of the 4R/6R genotype of the *DRD4* gene were more likely to be found amongst subjects with a severe level of anxiety and depression and the carriers of the *DRD4* allele 6R were more common among males diagnosed with depression. The number of tandem turns of the VNTR of the *DRD4* gene polymorphism was on the increase. The level of vital exhaustion also increased. The authors [23] claim that it is so because amongst people with the long allele of the *DRD4* exon 3 polymorphism, the affinity of dopamine with the receptor is reduced, and therefore, that group is less sensitive to dopamine. Hence, there is a high frequency of genotypes with the long allele of the *DRD4* exon 3 in males with anxiety, depression, and vital exhaustion. 

The same question was addressed by Bobadilla et al., who conducted their research to find out if the *DRD4* gene polymorphism was associated with a comorbidity in relation to depression and marijuana use symptoms in a large, diverse, non-clinical adolescent sample. In the study, they also explored how risk factors previously associated with substance use and depression (ethnicity, age, victimization, and alcohol-related problems) were linked to the co-occurrence of marijuana use and depression [24]. The ≥7R/≥7R *DRD4* genotype significantly heightened the risk of co-occurrent cannabis use and depressive symptoms, which was consistent with the hypothesis. As previously mentioned, a number of studies have linked the ≥7R allele to “less efficient functioning” at the molecular level [24].

Not only did we not confirm such a relationship, but we found something completely different in our study population. A statistically significant difference was shown in the frequency of *DRD4* exon 3 (Ex3) alleles between all patients diagnosed with PUD MDD and the controls, but the frequency of l alleles was lower in the PUD MDD group vs. controls (Table 3 (l 0.18 vs. 0.25, χ^2^ = 3.99, p = 0.046)).

Therefore, we included personality traits in our study. They are believed to play an important role in the case of psychiatric disorders. In various circumstances, each individual’s behavior follows particularly distinctive patterns. Personality traits are environmentally and biologically determined [25].

The aim of our study was to find possible associations between personality traits and *DRD4* exon 3 gene polymorphisms of male patients with polysubstance use disorder with cooccurrence of a depressive episode, which may present a specific endophenotype of this group of patients.

We found differences in personality traits between PUD MDD subjects, PUD subjects, and controls. PUD patients with a depressive episode delivered higher scores on the Neuroticism and Openness Scale and a higher level of anxiety (STAI trait/state scores) compared to patients with polysubstance use disorder without a depressive episode. Simultaneously, PUD MDD subjects delivered lower scores on the Agreeability and Conscientiousness Scales compared to PUD patients (Table 4). However, extraversion did not differentiate PUD MDD patients from PUD subjects, but it did differentiate both groups from controls (PUDD MD and PUD subjects had lower scores than controls). Our findings are consistent with the findings of other authors that people with substance use disorders have a common personality profile: high neuroticism, low conscientiousness, and low agreeableness [26,27,28]. In a Norwegian study, the opioid-dependent sample scored higher on neuroticism, lower on extraversion, and lower on conscientiousness compared to controls [29].

Thus, the main findings of our study are the interactions for the groups of PUD MDD subjects vs. controls between the *DRD4* genotype and two domains of the five-factor model of personality, i.e., extraversion and conscientiousness. The s/l heterozygous variants were linked to lower scores on the extraversion and conscientiousness scales—predisposed to be in the PUD MDD group (Figure 1 and Figure 2 and Table 7)—whereas the l/l homozygous carriers presented a higher level of extraversion and conscientiousness, predisposed to be in the control group. In our research, l/l alleles had a protective effect on the PUD MDD group.

This protective effect of the l alleles may be found in our post hoc analysis (Table 7). While in the PUDD MDD group, the mean sten score on the Extraversion Scale was 5.44 (Table 5), in the PUD MDD *DRD4* exon 3 (Ex3) l/l group, it was 9.0 (Table 7). The same happened for the Conscientiousness Scale: the mean sten score in the PUDD MDD group was 5.01, and in the PUD MDD *DRD4* exon 3 (Ex3) l/l group, it was 9.5. This effect is presented in Figure 1 and Figure 2. While examining interactions in the subjects with the *DRD4* exon 3 (Ex3) s/l polymorphism, a lower level of extraversion (sten scores) was found compared to the control group with the *DRD4* exon 3 (Ex3) s/l polymorphism. In contrast, an inverse correlation occurs for interactions in individuals with the *DRD4* exon 3 (Ex3) l/l polymorphism in the level of extraversion (for genotypic correlations in the control group, that level of extraversion did not change—as shown in Figure 1, no additive control + genetic polymorphism effect was found).

For interactions in the subjects (patients with polysubstance use disorder comorbid with a depressive episode) with the *DRD4* exon 3 (Ex3) s/l polymorphism, a lower level of conscientiousness (sten scores) may be found compared to the control group with the *DRD4* exon 3 (Ex3) s/l polymorphism (4.39 vs. 6.13). In contrast, an inverse correlation occurs for interactions in the subjects with the *DRD4 e*xon 3 (Ex3) l/l polymorphism. Their level of conscientiousness (sten scores) is higher than in the control group with the *DRD4* exon 3 (Ex3) l/l polymorphism (9.5 vs. 6.15; F_2,389_ = 5.24, *p* = 0.006). Furthermore, the level of conscientiousness in the control group did not change depending on the variant of the *DRD4* exon 3 (Ex3) s/l polymorphism (no additive control + genetic polymorphism effect was found) (Figure 2).

Conscientiousness includes good impulse control and goal-directed behaviors, whereas extraversion means talkativeness and assertiveness. A person with low scores on the Extraversion Scale is an introvert. Some studies have documented the association between *DRD4* and temperament, or personality traits. Their results suggest that the long allele is linked to high novelty seeking and risk taking, constricted emotional responses, and with preserved attention processing of emotional stimuli and efficient problem solving. This may explain its protective effect observed in our study.

In three high-impact consecutive publications, the long allele of the *DRD4* exon 3 polymorphism was correlated with a significant increase in the frequency of novelty seeking [12,13,30]. Novelty seeking correlates positively with extraversion and negatively with conscientiousness; therefore, associations between long *DRD4* exon 3 alleles and higher extraversion and lower conscientiousness scores were reported respectively using the NEO-PI-R scales [25]. This polymorphism has its effect on exploratory, extraverted, rather than impulsive subtypes of novelty seeking. A number of research studies provided no support for this hypothesis on the association between polymorphisms of the dopamine D4 receptor gene, novelty seeking, and personality [31,32,33,34].

However, one should draw no definitive conclusions and be aware of the limitations of the study—i.e., the size of the group and the fact that the genetic aspect was only partially analyzed. In the case of multi-genetic and multi-factorial disorders that we have studied, it is difficult to draw any firm conclusions. Nevertheless, we hope our analysis broadens the view on the biological aspects of these disorders.

## 5. Conclusions

In conclusion, the comparison of patients diagnosed with polysubstance use disorder comorbid with a depressive episode to the control group of healthy volunteers shows that the former delivered significantly higher scores on both the STAI state and trait scale and the NEO-FFI Neuroticism and Openness Scale, and lower scores on the Extraversion, Agreeability, and Conscientiousness NEO-FFI Scales. In the *DRD4* exon 3 (Ex3) gene polymorphism, the s allele was more frequent in the study group, and the l allele was less frequent. In our research study, l/l alleles exerted a protective effect on the PUD MDD group. This protective effect was mediated by the fact that the l/l homozygous carriers had a higher level of extraversion and conscientiousness. Our findings contradict a number of studies which confirmed associations between the 7R and an increased risk of various neuropsychiatric disorders, including depression and anxiety. This contradiction may suggest that it is better to conceptualize the *DRD4* gene as a plasticity gene whose effect may point to either positive or negative dependence on particular environments. According to Jiang, the role of D4 receptor gene exon 3 polymorphisms is to shape a prosocial behavior and altruism. Differential susceptibility alleles may be more prosocial if they are influenced by one environment, and less prosocial in another [35].

These associations reveal that psychological factors combined with genetic data identify a new area of research on addiction, including the problem of diagnosis. However, we want to be careful and draw no definite conclusions at this stage of our research.

## Figures and Tables

**Table 1:** Type of psychoactive substance use in addicts.

Type of Substance/Addiction	All Patients Diagnosed with PUD MDD (*n* = 95)	
	*n*	%
Behavioral addiction	43	45.3
Designer drugs	21	22.1
F10.2—alcohol	56	58.9
F11.2—opiates	21	22.1
F12.2—cannabinols	69	72.6
F13.2—sedatives and hypnotics	14	14.7
F14.2—cocaine	8	8.4
F15.2—stimulants	78	82.1
F16.2—hallucinogenic	13	13.7
F19.2—mixed addictions	60	63.2

**Table 2:** Hardy–Weinberg’s law for patients with polysubstance use disorder comorbid with a depressive episode (PUD MDD), for patients diagnosed with polysubstance use disorder (PUD) and control subjects.

Hardy–Weinberg Equilibrium Calculator Including Analysis for Ascertainment Bias		Observed (Expected)		Test χ^2^	
				χ^2^	*p*
*DRD4* Ex3 PUD MDD	s/s	63 (64)	l allele freq = 0.18 s allele freq = 0.82	0.529	>0.05
	s/l	30 (27.9)			
	l/l	2 (3)			
*DRD4* Ex3 PUD	s/s	127 (128.2)	l allele freq = 0.21 s allele freq = 0.79	0.245	>0.05
	s/l	71 (68.6)			
	l/l	8 (9.2)			
*DRD4* Ex3 control subjects	s/s	177 (169.7)	l allele freq = 0.25 s allele freq = 0.75	5.075	<0.05
	s/l	98 (112.6)			
	l/l	26 (18.7)			

*p*, statistical significance χ^2^ test; s, short; l, long.

**Table 3:** Frequency of genotypes of *DRD4* exon 3 (Ex3) gene polymorphisms in patients diagnosed with PUD MDD, patients diagnosed with PUD, and control subjects.

Group	*DRD4* Ex3				
	Genotypes			Alleles	
	s/s *n* (%)	s/l *n* (%)	l/l *n* (%)	s *n* (%)	l *n* (%)
A: PUD MDD *n* = 95	63 (66)	30 (32)	2 (2)	156 (82)	34 (18)
B: PUD *n* = 206	127 (62)	71 (34)	8 (4)	325 (79)	87 (21)
C: Control *n* = 301	177 (59)	98 (33)	26 (9)	452 (75)	150 (25)
χ^2^ (*p* value)	A/B: 1.01 (0.605) A/C: 5.05 (0.079) B/C: 4.42 (0.110)			A/B: 0.84 (0.359) A/C: 3.99 (0.046 *) B/C: 1.970 (0.160)	

*n*, number of subjects; * statistically significant differences.

**Table 4:** STAI and NEO Five Factor Inventory sten scores between healthy controls and all patients diagnosed with polysubstance use disorder comorbid with a depressive episode (PUD MDD) and all patients diagnosed with polysubstance use disorder comorbid with a depressive episode (PUD).

STAI/NEO Five Factor Inventory/	A: PUD MDD (*n* = 95)	B: PUD (*n* = 206)	C: Control (*n* = 301)	A/C: Z *(p-*Value)	B/C: Z (*p-*Value)	A/B: *Z* *(p-*Value*)*
STAI trait/scale	7.62 ± 2.25	6.87 ± 2.25	5.16 ± 2.17	7.062 (0.0000 *)	7.768 (0.0000 *)	3.665 (0.0002 *)
STAI state/scale	6.65 ± 2.21	5.54 ± 2.43	4.68 ± 2.14	8.207 (0.0000 *)	4.162 (0.0000 *)	2.754 (0.0059 *)
Neuroticism/scale	7.34 ± 2.00	6.45 ± 2.20	4.67 ± 2.01	9.264 (0.0000 *)	8.683 (0.0000 *)	3.094 (0.0020 *)
Extraversion/scale	5.44 ± 2.36	5.90 ± 2.01	6.37 ± 1.97	−3.640 (0.0003 *)	−2.412 (0.0147 *)	−1.837 (0.0602)
Openness/scale	5.46 ± 2.01	4.80 ± 2.00	4.53 ± 1.61	4.136 (0.0000 *)	1.401 (0.1545)	2.557 (0.0106 *)
Agreeability/scale	4.05 ± 1.99	4.41 ± 1.90	5.60 ± 2.09	−6.286 (0.0000 *)	−6.165 (0.0000 *)	−1.959 (0.0501 *)
Conscientiousness/scale	5.01 ± 2.14	5.85 ± 2.29	6.07 ± 2.15	−4.102 (0.0000 *)	−1.014 (0.3059)	−3.006 (0.0027 *)

*p*, statistical significance with Mann–Whitney U-test; *n,* number of subjects; M ± SD, mean ± standard deviation; * statistically significant differences.

**Table 5:** Differences in *DRD4* exon 3 (*DRD4* Ex3), the NEO Five Factor Inventory, and the STAI scale between healthy controls and PUD MDD subjects.

STAI/NEO Five Factor Inventory	DRD4 Ex3					ANOVA			
	PUD MDD (*n* = 95)	Control (*n* = 301)	s/s (*n* = 240)	s/l (*n* = 127)	l/l (*n* = 28)	Factor	F (*p*-Value)	ɳ^2^	Power (Alfa = 0.05)
STAI trait/scale	7.62 ± 2.25	5.16 ± 2.17	5.72 ± 2.54	5.87 ± 2.21	5.32 ± 2.34	intercept	F_1,389_ = 499.54 (*p* < 0.0001) *	0.563	1.000
						PUD MDD/control	F_1,389_ = 17.06 (*p* < 0.000) *	0.042	0.985
						*DRD4* Ex3	F_2,389_ = 0.02 (*p* = 0.976)	0.0001	0.054
						PUD MDD/control × *DRD4* Ex3	F_2,389_ = 0.64 (*p* = 0.527)	0.003	0.157
STAI state/scale	6.65 ± 2.21	4.68 ± 2.14	5.10 ± 2,33	5.39 ± 2.24	4.61 ± 2.39	intercept	F_1,389_ = 386.18 (*p* < 0.0001) *	0.498	1.000
						PUD MDD/control	F_1,389_ = 8.40 (*p* = 0.004) *	0.021	0.824
						*DRD4* Ex3	F_2,389_ = 0.88 (*p* = 0.415)	0.004	0.201
						PUD MDD/control × *DRD4* Ex3	F_2,389_ = 0.32 (*p* = 0.729)	0.002	0.100
Neuroticism/scale	7.34 ± 2.00	4.67 ± 2.01	5.31 ± 2.27	5.45 ± 2.32	4.50 ± 2.51	intercept	F_1,389_ = 515.96 (*p* < 0.0001) *	0.571	1.000
						PUD MDD/control	F_1,389_ = 26.51 (*p* < 0.0001) *	0.064	0.999
						*DRD4* Ex3	F_2,389_ = 0.73 (*p* = 0.480)	0.004	0.174
						PUD MDD/control × *DRD4* Ex3	F_2,389_ = 0.04 (*p* = 0.954)	0.0002	0.057
Extraversion/scale	5.44 ± 2.36	6.37 ± 1.97	6.20 ± 2.04	5.90 ± 2.19	6.89 ± 2.11	intercept	F_1,389_ = 588.12 (*p* < 0.0001) *	0.602	1.000
						PUD MDD/control	F_1,389_ = 0.003 (*p* = 0.953)	0.00001	0.050
						*DRD4* Ex3	F_2,389_ = 6.23 (*p* = 0.002) *	0.031	0.893
						PUD MDD/control × *DRD4* Ex3	F_2,389_ = 4.22 (*p* = 0.015) *	0.021	0.738
Openness/scale	5.46 ± 2.01	4.53 ± 1.61	4.64 ± 1.77	4.96 ± 1.74	4.75 ± 1.67	intercept	F_1,389_ = 568.45 (*p* < 0.0001) *	0.594	1.000
						PUD MDD/control	F_1,389_ = 9.83 (*p* = 0.002) *	0.024	0.879
						*DRD4* Ex3	F_2,389_ = 1.66 (*p* = 0.190)	0.008	0.350
						PUD MDD/control × *DRD4* Ex3	F_2,389_ = 0.88 (*p* = 0.416)	0.004	0.201
Agreeability/scale	4.05 ± 1.99	5.60 ± 2.09	5.12 ± 2.21	5.41 ± 2.12	5.36 ± 1.97	intercept	F_1,389_ = 313.18 (*p* < 0.0001) *	0.447	1.000
						PUD MDD/control	F_1,389_ = 9.69 (*p* = 0.002)	0.024	0.874
						*DRD4* Ex3	F_2,389_ = 2.37 (*p* = 0.554)	0.003	0.142
						PUD MDD/control × *DRD4* Ex3	F_2,389_ = 0.30 (*p* = 0.071)	0.0004	0.061
Conscientiousness/scale	5.01 ± 2.14	6.07 ± 2.15	5.80 ± 2.21	5.75 ± 2.18	6.39 ± 2.10	intercept	F_1,389_ = 510.13 (*p* < 0.0001) *	0.568	1.000
						PUD MDD/control	F_1,389_ = 0.18 (*p* = 0.668)	0.0005	0.071
						*DRD4* Ex3	F_2,389_ = 5.09 (*p* = 0.007) *	0.026	0.820
						PUD MDD/control × *DRD4* Ex3	F_2,389_ = 5.24 (*p* = 0.006) *	0.026	0.831

* Significant result.

**Table 6:** Differences in *DRD4* exon 3 (*DRD4* Ex3), the NEO Five Factor Inventory, the STAI scale between healthy controls and PUD subjects.

STAI/NEO Five Factor Inventory	DRD4 Ex3					ANOVA			
	PUD (*n* = 206)	Control (*n* = 301)	s/s (*n* = 304)	s/l (*n* = 169)	l/l (*n* = 34)	Factor	F (*p*-Value)	ɳ^2^	Power (Alfa = 0.5)
STAI trait/scale	6.87 ± 2.25	5.16 ± 2.17	5.83 ± 2.49	6.00 ± 2.16	5.29 ± 2.14	intercept	F_1,501_ = 1255.43 (*p* < 0.0001) *	0.716	1.000
						PUD/control	F_1,501_ = 15.92 (*p* < 0.0001) *	0.031	0.978
						*DRD4* Ex3	F_2,501_ = 0.98 (*p* = 0.375)	0.004	0.221
						PUD/control x *DRD4* Ex3	F_2,501_ = 1.52 (*p* = 0.219)	0.006	0.324
STAI state/scale	5.54 ± 2.43	4.68 ± 2.14	4.99 ± 2.30	5.21 ± 2.27	4.50 ± 2.40	intercept	F_1,501_ = 849.36 (*p* < 0.0001) *	0.630	1.000
						PUD/control	F_1,501_ = 2.14 (p = 0.144)	0.004	0.309
						*DRD4* Ex3	F_2,501_ = 1.41 (*p* = 0.245)	0.006	0.303
						PUD/control x *DRD4* Ex3	F_2,501_ = 1.21 (*p* = 0.299)	0.005	0.264
Neuroticism/scale	6.45 ± 2.20	4.67 ± 2.01	5.46 ± 2.29	5.39 ± 2.16	4.67 ± 2.43	intercept	F_1,501_ = 1196.67 (*p* < 0.0001) *	0.705	1.000
						PUD/control	F_1,501_ = 27.48 (*p* < 0.0001) *	0.052	0.999
						*DRD4* Ex3	F_2,501_ = 0.82 (*p* = 0.442)	0.003	0.190
						PUD/control x *DRD4* Ex3	F_2,501_ = 1.31 (*p* = 0.270)	0.005	0.284
Extraversion/scale	5.90 ± 2.01	6.37 ± 1.97	6.15 ± 1.98	6.11 ± 2.03	6.76 ± 2.05	intercept	F_1,501_ = 1803.32 (*p* < 0.0001) *	0.782	1.000
						PUD/control	F_1,501_ = 0.73 (*p* = 0.391)	0.001	0.137
						*DRD4* Ex3	F_2,501_ = 1.45 (*p* = 0.234)	0.005	0.311
						PUD/control x *DRD4* Ex3	F_2,501_ = 0.34 (*p* = 0.713)	0.001	0.104
Openness/scale	4.80 ± 2.00	4.53 ± 1.61	4.51 ± 1.74	4.84 ± 1.82	4.85 ± 1.89	intercept	F_1,501_ = 1335.71 (*p* < 0.0001) *	0.727	1.000
						PUD/control	F_1,501_ = 3.99 (*p* = 0.046)	0.008	0.514
						*DRD4* Ex3	F_2,501_ = 2.61 (*p* = 0.0740)	0.010	0.520
						PUD/control x *DRD4* Ex3	F_2,501_ = 0.99 (*p* = 0.372)	0.003	0.222
Agreeability/scale	4.41 ± 1.90	5.60 ± 2.09	5.09 ± 2.12	5.22 ± 2.00	4.82 ± 2.25	intercept	F_1,501_ = 993.33 (*p* < 0.0001) *	0.665	1.000
						PUD/control	F_1,501_ = 33.04 (*p* < 0.0001) *	0.061	0.999
						*DRD4* Ex3	F_2,501_ = 2.95 (*p* = 0.053)	0.012	0.574
						PUD/control x *DRD4* Ex3	F_2,501_ = 2.30 (*p* = 0.101)	0.009	0.468
Conscientiousness/scale	5.85 ± 2.29	6.07 ± 2.15	5.93 ± 2.27	6.01 ± 2.14	6.26 ± 1.94	intercept	F_1,501_ = 1357.97 (*p* < 0.0001) *	0.730	1.000
						PUD/control	F_1,501_ = 0.002 (*p* = 0.961)	0.00001	0.050
						*DRD4* Ex3	F_2,501_ = 0.53 (*p* = 0.587)	0.002	0.138
						PUD/control x *DRD4* Ex3	F_2,501_ = 0.31 (*p* = 0.729)	0.001	0.100

* Significant result.

**Table 7:** Post hoc analysis of interactions between patients with polysubstance use disorder comorbid with a depressive episode (PUD MDD)/control and *DRD4* exon 3 (*DRD4* Ex3) and the NEO FFI Extraversion/Conscientiousness Scale.

***DRD4*** **Ex3 and NEO FFI Extraversion Scale**						
	**{1}** **M = 4.57**	**{2}** **M = 5.71**	**{3}** **M = 9.00**	**{4}** **M = 6.28**	**{5}** **M = 6.37**	**{6}** **M = 6.73**
PUD MDD *DRD4* Ex3 s/l {1}		0.0145 *	0.0033 *	0.0001 *	<0.0000 *	0.0001 *
PUD MDD *DRD4* Ex3 s/s {2}			0.0261 *	0.0905	0.0290 *	0.0339 *
PUD MDD *DRD4* Ex3 l/l {3}				0.0633	0.0720	0.1319
control *DRD4* Ex3 s/l {4}					0.7060	0.3143
control *DRD4* Ex3 s/s {5}						0.4060
control *DRD4* Ex3 l/l {6}						
***DRD4*** **Ex3 and NEO FFI Conscientiousness Scale**						
	**{1}** **M = 4.39**	**{2}** **M = 5.14**	**{3}** **M = 9.50**	**{4}** **M = 6.13**	**{5}** **M = 6.04**	**{6}** **M = 6.15**
PUD MDD *DRD4* Ex3 s/l {1}		0.1210	0.0011 *	0.0002 *	0.0002 *	0.0025 *
PUD MDD *DRD4* Ex3 s/s {2}			0.0045 *	0.0041 *	0.0042 *	0.0419 *
PUD MDD *DRD4* Ex3 l/l {3}				0.0271 *	0.0226 *	0.0325 *
control *DRD4* Ex3 s/l {4}					0.7281	0.9640
control *DRD4* Ex3 s/s {5}						0.7980
control *DRD4* Ex3 l/l {6}						

* Statistically significant differences; M, mean.

## Data Availability

Not applicable.
